# Slow resolution of inflammation in severe adult dengue patients

**DOI:** 10.1186/s12879-016-1596-x

**Published:** 2016-06-14

**Authors:** Lingzhai Zhao, Xiuyan Huang, Wenxin Hong, Shuang Qiu, Jian Wang, Lei Yu, Yaoying Zeng, Xinghua Tan, Fuchun Zhang

**Affiliations:** Guangzhou Eighth People’s Hospital, Guangzhou Medical University, Guangzhou, Guangdong 510060 China; Department of Immunobiology, Jinan University, Guangzhou, Guangdong 510632 China

**Keywords:** Severe dengue, Resolution of inflammation, Cytokine, Chemokine, Adhesion molecule

## Abstract

**Background:**

The pathogenesis of severe dengue has not been fully elucidated. The inflammatory response plays a critical role in the outcome of dengue disease.

**Methods:**

In this study, we investigated the levels of 17 important inflammation mediators in plasma collected from mild or severe adult dengue patients at different time points to understand the contribution of inflammation to disease severity and to seek experimental evidence to optimize the existing clinical treatment strategies. Patients were simply classified as mild and severe dengue according to the 2009 WHO classification. Plasma was collected on day 3-5, 6-7, 8-10 and 14-17 of illness. Levels of 17 inflammation mediators including TNF-α, IL-1α, IFN-γ, IL-6, IFN-α, MIF, IL-10, IL-1RA, IL-8, IP-10, MCP-1, RANTES, GRO, eotaxin-1, sICAM-1 and sVCAM-1 were determined by a multiplex Luminex® system. Different trends of inflammation mediators throughout the disease were compared between mild and severe patients.

**Results:**

Inflammation mediators including IL-1α, IFN-γ, IL-10, IL-8, IP-10, MCP-1 and sVCAM-1 displayed significant differences on day 8-10 of illness between mild and severe dengue patients. Their concentrations were higher in severe patients than mild ones at the same time points. Moreover, those cytokines decreased gradually in mild patients but not in severe patients.

**Conclusion:**

Our results revealed the coexistence of excessive inflammatory response and slow resolution of inflammation in severe adult dengue patients. Hence suppression and/or pro-resolution of inflammation could be a potential therapeutic approach for treatment of severe dengue.

## Background

Dengue, caused by dengue virus (DENV) infection, is considered as the most prevalent mosquito-borne viral disease in humans. An estimated 390 million individuals are infected annually by DENV in tropical and subtropical regions [1]. All four serotypes of DENV (DENV-1 to DENV-4) can cause a spectrum of illness ranging from asymptomatic to life-threatening dengue shock syndrome [2]. Symptomatic disease includes two entities: mild and severe dengue according to the 2009 World Health Organization (WHO) classification [3]. Mild dengue is a self-limited illness lasting about 7 days. However, severe dengue, characterized by plasma leakage, may be fatal. Although many efforts have been made to elucidate the underlying mechanisms of severe disease, it still remains unclear [4, 5].

The inflammatory response against DENV is believed to play an important role in its pathogenesis [6]. The different manifestations between mild and severe dengue patients indicate that inflammatory response may differ substantially. Many studies have demonstrated that levels of inflammation mediators such as TNF-α, IFN-γ, IP-10, IL-8 are elevated in dengue patients and higher levels in severe cases [7–9]. It has been suggested that inflammation plays a critical role in the outcome of DENV infection. The inflammation is beneficial in providing protection against infection and normally terminated once the infection is cleared, while it is detrimental when dysregulated [10, 11]. Similar to the initiation of inflammation, the resolution of inflammation would be crucial for a successful outcome [12].

Dengue is an expanding public threat to people in South China, where the patients exhibit different symptoms comparative to Southeast Asia. In Southeast Asia, children have greater risk of severe disease while adults show a mild disease. In 2013, China experienced a large dengue outbreak after 1990s, which was especially prevalent in Xishuangbanna region in Yunnan province where an autochthonous dengue outbreak has not been recorded in the last decade and Guangzhou city in Guangdong province where both incidence and case numbers are the highest among Chinese cities [13]. A total of 4432 cases were reported and 86 were severe ones, with an incidence rate of severe dengue of 1.9 % [14]. In this study, we determined levels of 17 important inflammation mediators in plasma collected from mild or severe adult dengue patients at different time points to understand the contribution of inflammation to disease severity and to seek experimental evidence to optimize the existing clinical treatment strategies.

## Methods

### Study subjects and samples

Fifty one dengue patients and 10 healthy volunteers were included in this study from Guangzhou Eighth People’s Hospital and Xishuangbanna Dai Autonomous Prefecture People’s Hospital between August 20 and November 15 in 2013. All patients were laboratory confirmed to have dengue by dengue virus specific antibody test and real-time PCR assay using serum samples obtained during acute phase of infection. Patients were classified as mild and severe dengue following the 2009 WHO dengue classification. With any of the following conditions, cases were diagnosed as severe disease: (1) plasma leakage leading to shock, respiratory distress, or both; (2) severe bleeding; and (3) severe organ impairment. Plasma of 51 patients was collected on day 3–5, 6–7, 8–10 and 14–17 of illness. Plasma was collected from 10 age and gender matched healthy volunteers as controls. All samples were stored at −80 °C. Data on demographic characteristics, clinical features and routine laboratory test findings were also collected.

### Diagnosis of primary or secondary infection

Dengue IgM and IgG capture ELISA kits (Panbio, Brisbane, Queensland, Australia) were used to diagnose primary or secondary dengue infection according to the manufacturer’s protocol using serum during acute stage. Dengue-specific IgM positive only was indicative of primary infection. Both IgM and IgG positive or IgG positive only was diagnosed as secondary infection.

### Serotype of DENV assay

Serotypes of DENV were determined by serotype specific fluorescent PCR diagnostic kits (DAAN Ltd, Guangzhou, Guangdong, China) according to the manufacturer’s protocol and performed on an ABI 7500 real-time PCR system (ABI, Foster city, CA).

### Measurement of inflammation mediators

Inflammation mediators were measured using MILLIPLEX® MAP human cytokine/chemokine panel 1 kit and human sepsis panel 1 kit (Merck Millipore, Germany) according to the manufacturer’s instructions. Briefly, patients’ plasma was mixed with beads coated with capture antibodies to various mediators. Then the mixtures were incubated with biotinylated detection antibodies. Finally, PE-conjugated streptavidin was added, and the fluorescent signals were detected using Luminex® 200™ System (Life Technologies, Grand Island, NY). Raw data was initially measured as the relative fluorescence intensity and then converted to concentration based on the standard curves generated from the reference concentrations supplied in the kits. 17 inflammation mediators were measured including tumor necrosis factor-α (TNF-α), interleukin-1α (IL-1α), interferon-γ (IFN-γ), interleukin-6 (IL-6), interferon-α (IFN-α), macrophage migration inhibitory factor (MIF), interleukin-10 (IL-10), interleuekin-1 receptor antagonists (IL-1RA), interleukin-8 (IL-8), interferon-inducible protein-10 (IP-10), monocyte chemotactic protein-1 (MCP-1), regulated upon activation normal T-cell expressed and secreted (RANTES), growth related oncogene-α (GRO-α), eotaxin-1, soluble intercellular adhesion molecule-1 (sICAM-1) and soluble vascular cell adhesion molecule-1 (sVCAM-1).

### Statistical analysis

Data are reported as mean ± standard deviation (SD) for clinical parameters. Statistical analysis was done using GraphPad Prism 5.0 (GraphPad software, San Diego, USA) by *t* tests or non-parametric tests. Differences of *P < 0.05* were considered statistically significant.

## Results

### Characteristics of dengue patients

Fifty one dengue patients were included in this study. 30 were diagnosed as mild dengue and 21 were severe ones. Detailed demographic, epidemiologic and clinical features of patients are summarized in Tables 1 and 2. All patients were adults. Age was between 18 to 61 years old. Laboratory tests on peripheral blood examination were summarized in Table 2. Results showed that leukopenia especially neutropenia and thrombocytopenia occurred in all patients but no increased hematocrit. Albumin was decreased, while alanine aminotransferase (ALT), aspartate aminotransferase (AST), creatine kinase (CK), lactate dehydrogenase (LDH) increased in all patients. Platelet (PLT) was lower (*P = 0.048*) while AST was higher (*P = 0.021)* in severe patients than mild ones.

**Table 1:** Demographic and epidemiologic features of mild and severe dengue patients

Items	Mild (*n* = 30)	Severe (*n* = 21)
Age, median (range)	35 (18-59)	39(18-61)
Gender		
Male, no. (%)	21 (70)	7 (33.3)
Female, no. (%)	9 (30)	14 (66.7)
Primary or secondary infection		
Primary infection, no. (%)	21 (70)	11 (52.4)
Secondary infection, no. (%)	9 (30)	10 (47.6)
DENV serotype		
DENV-1, no. (%)	22 (73.3)	10 (47.6)
DENV-3, no. (%)	8 (26.7)	11 (52.4)

**Table 2:** Clinical features and laboratory data from mild and severe dengue patients

Items	Mild (*n* = 30)	Severe (*n* = 21)
Clinical features (%)		
Fever	100	100
Rash	53.3	71.4
Myalgia or Arthralgia	63.3	52.4
Hypodynamia	50	100
Vomiting	16.7	28.6
Abdominal pain	0	33.3
Diarrhea	3.3	19
Jaundice	0	14.3
Splenomegalia	10	23.8
Positive tourniquet test	Not detected	94
Haemorrhagia	0	61.9
Plasma leakage	0	33.3
Hypotension	0	28.6
Laboratory data (Mean ± SD)		
White Blood Cells count (×10^9^/L)	3.7 ± 1.5	3.7 ± 2.1
Neutrophils count (×10^9^/L)	1.9 ± 1.6	1.4 ± 0.8
Lymphocytes count (×10^9^/L)	1.3 ± 0.7	1.8 ± 1.9
Monocytes count (×10^9^/L)	0.4 ± 0.2	0.4 ± 0.2
CD3^+^T cells count (cells/μl)	1026 ± 552	957 ± 592
CD4^+^T cells count (cells/μl)	511 ± 280	493 ± 271
CD8^+^T cells count (cells/μl)	466 ± 322	442 ± 379
CD4/CD8 ratio	1.26 ± 0.80	1.37 ± 0.68
Hematocrit (HCT, %)	42 ± 9	39 ± 6
Platelet (×10^9^/L)	75 ± 56	48 ± 33*
Total Billirubin (μmol/L)	12.19 ± 5.03	13.85 ± 7.90
Total Protein (g/L)	65 ± 6	62 ± 6
Albumin (g/L)	39 ± 4	36 ± 4
Alanine Transaminase (ALT, U/L)	60 ± 71	89 ± 60
Aspartate Transaminase (AST, U/L)	77 ± 65	126 ± 71*
Alkaline Phosphatase (ALP, U/L)	65 ± 23	96 ± 103
Creatine kinase (CK, U/L)	203 ± 140	231 ± 185
Lactate Dehydrogenase (LDH, U/L)	369 ± 165	490 ± 271
Creatinine (μmol/L)	79 ± 17	75 ± 43

*P < 0.05, severe patients *vs* mild patients

### Levels of inflammatory mediators and dynamic of inflammation resolution at different time points in mild and severe dengue patients

Levels of 17 inflammation mediators including 6 pro-inflammatory cytokines, 2 anti-inflammatory cytokines, 7 chemokines and 2 adhesion molecules were determined in mild and severe dengue patients at four time points, on day 3–5, 6–7, 8–10 and 14–17 of disease to observe trends of the mediators over the course of disease. We chose to analyze levels of these mediators on day 6–7 and 8–10 of illness in order to understand the difference in inflammation resolution between mild and severe dengue patients.

#### Pro-inflammatory cytokines

**Fig. 1:**
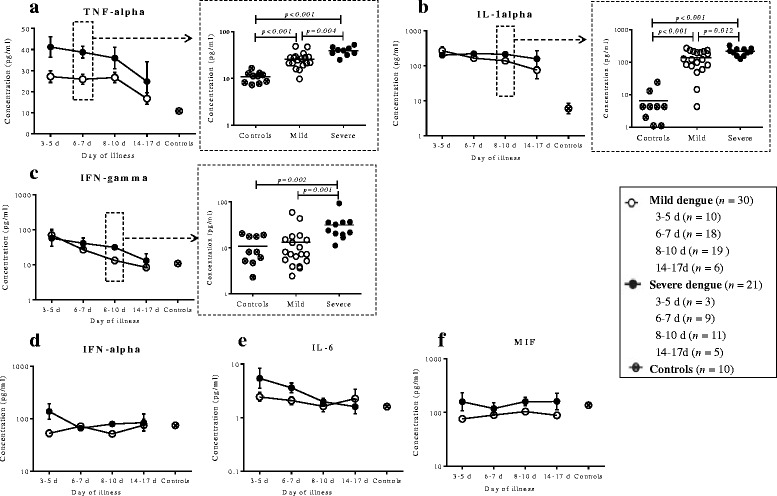
Dynamic changes of pro-inflammatory cytokines were compared in dengue patients. TNF-α (**a**), IL-1α (**b**) and IFN-γ (**c**) were significantly elevated in dengue patients. Then they decreased as patients were recovery. Moreover, higher level of TNF-α on day 6-7 and higher levels of IL-1α and IFN-γ on day 8-10 of illness were observed in severe dengue patients than mild ones. However, IFN-α (**d**), IL-6 (**e**) and MIF (**f**) did not display significant abnormality at each time point. In addition, no differences of IFN-α, IL-6 and MIF were observed on day 6-7 and on day 8-10 of illness between mild and severe dengue patients

Six pro-inflammatory cytokines, including TNF-α, IL-1α, IFN-γ, IL-6, IFN-α and MIF, were determined. As Fig. 1 shown, concentrations of TNF-α {*P* < 0.01 (Day 3-5), *P* < 0.01 (Day 6-7), *P* < 0.01 (Day 8-10), Fig. 1a}, IL-1α {*P* = 0.003 (Day 3–5), *P* < 0.01 (Day 6–7), *P* < 0.01 (Day 8–10), Fig. 1b} and IFN-γ {*P* = 0.047 (Day 3–5), *P* = 0.074 (Day 6–7), *P* = 0.161(Day 8–10), Fig. 1c} were significantly elevated in dengue patients compared to healthy controls at early stage of illness. Then these cytokines exhibited a decreasing trend as patients recovered {TNF-α: *P* = 0.058 *versus* Controls (Day 14–17); IL-1α: *P* = 0.094 *versus* Controls (Day 14–17); IFN-γ: *P* = 0.919 *versus* Controls (Day 14–17)}. Moreover, higher level of TNF-α (*P* =0.004) on day 6–7 and higher levels of IL-1α (*P* = 0.012) and IFN-γ (*P* = 0.001) on day 8–10 of illness were observed in severe dengue patients than mild ones. However, IFN-α (*P* > 0.05, Fig. 1d), IL-6 (*P* > 0.05, Fig. 1e) and MIF (*P* > 0.05, Fig. 1f) did not display significant abnormality at each time point. In addition, no differences of IFN-α (*P* > 0.05, Fig. 1d), IL-6 (*P* > 0.05, Fig. 1e) and MIF (*P* > 0.05, Fig. 1f) were observed on day 6–7 and on day 8–10 of illness between mild and severe dengue patients.

#### Anti-inflammatory cytokines

**Fig. 2:**
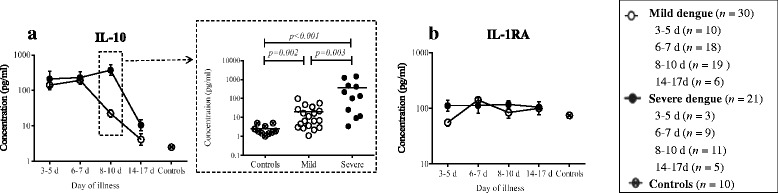
Different trends of anti-inflammatory cytokines were observed between mild and severe dengue patients. IL-10 was increased at early stage of illness in dengue patients. However, it showed different decreasing patterns between mild and severe patients (**a**). No abnormality was observed with regard to IL-1RA at each time points between dengue patients and healthy controls (**b**)

IL-10, as one of the important anti-inflammatory cytokines, was increased at early stage of illness in dengue patients than healthy controls {*P* = 0.008 (Day 3–5), *P* = 0.001 (Day 6–7), *P* = 0.027 (Day 8–10), *P* = 0.098 (Day 14–17), Fig. 2a}. However, it showed different decreasing patterns between mild and severe patients (Fig. 2a). Mild dengue patients displayed decreased trends after day 6–7 of illness (Fig. 2a). In contrast, increased trends were observed in severe dengue patients (Fig. 2a). The concentration of IL-10 was significantly higher in severe than mild patients on day 8–10 of illness (*P* = 0.003, Fig. 2a). No significant difference was observed between dengue patients and healthy controls with respect to plasmatic IL-1RA concentration (*P* > 0.05, Fig. 2b).

#### Chemokines

**Fig. 3:**
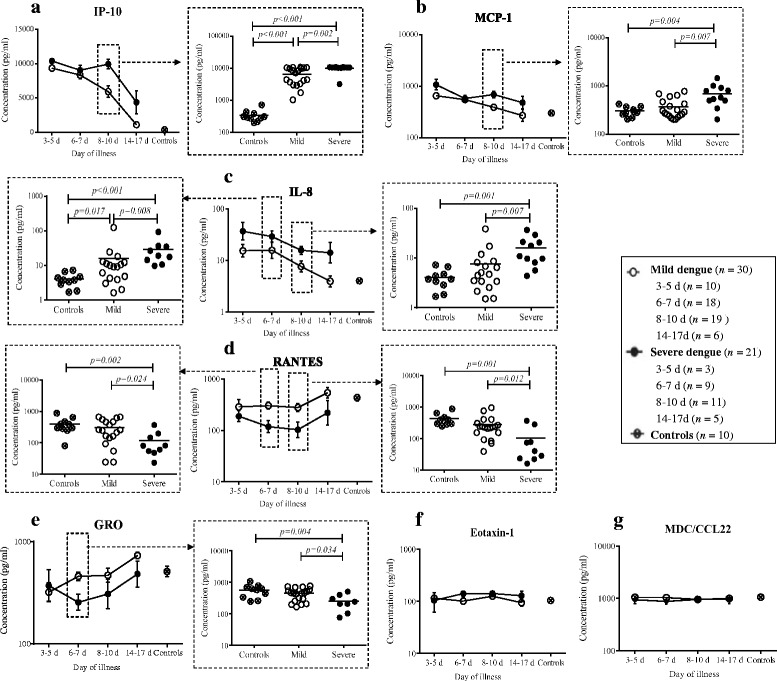
Different trends of chemokines throughout the disease were exhibited between mild and severe dengue patients. IP-10 (**a**) and MCP-1 (**b**) were higher in dengue patients compared with healthy controls. They displayed similar decreasing trends throughout the whole disease process. Significant differences of IP-10 and MCP-1 were noted between mild and severe dengue patients on day 8-10, with continually decreasing trends in mild patients but not in severe patients. IL-8 (**c**) was higher in severe patients than mild patients and showed decreasing trends as patients recovered. Levels of RANTES (**d**) and GRO-α (**e**) were lower in severe dengue patients. No difference of eotaxin-1 (**f**) and MDC (**g**) in dengue patients was observed compared to healthy controls

Seven chemokines, including IL-8, IP-10, MCP-1, RANTES, GRO-α, eotaxin-1 and MDC, were detected. IL-8 was higher in severe patients than mild patients and showed decreasing trends as patients recovered {(*P =* 0.008 (Day 6–7), *P =* 0.007 (Day 8–10), Fig. 3c}. IP-10 {*P* < 0.01 (Day 3–5), *P* < 0.01 (Day 6–7), *P* < 0.01 (Day 8–10), *P* = 0.037 (Day 14–17), Fig. 3a} and MCP-1 {*P* < 0.01 (Day 3–5), *P* < 0.01 (Day 6–7), *P* = 0.040 (Day 8–10), *P* = 0.500 (Day 14–17), Fig. 3b} were higher in dengue patients compared with healthy controls. They displayed similar decreasing trends throughout the disease progress. Significant differences of IP-10 (*P* = 0.002, Fig. 3a) and MCP-1 (*P = 0.007*, Fig. 3b) were noted between mild and severe dengue patients on day 8–10, with continually decreasing trends in mild patients but not in severe patients. Level of RANTES was lower in severe dengue patients compared to mild patients and healthy controls on day 6–7 (*P* = 0.002 *versus* healthy controls, *P* = 0.024 *versus* mild patients) and 8–10 (*P* = 0.001 *versus* healthy controls, *P* = 0.012 *versus* mild patients) of illness (Fig. 3d). GRO-α was similar to RANTES but a significant difference was noted only on day 6–7 of illness between mild and severe dengue patients (*P* = 0.034, Fig. 3e). No difference was observed between dengue patients and healthy controls in eotaxin-1 (*P* > 0.05, Fig. 3f) and MDC (*P* > 0.05, Fig. 3g).

#### Adhesion molecules

**Fig. 4:**
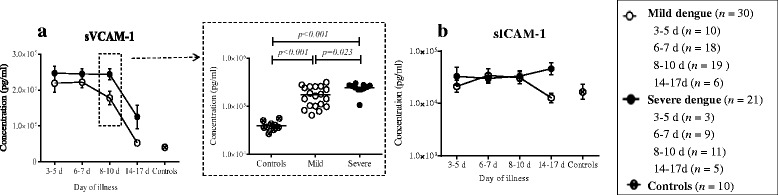
Different trends of adhesion molecules were compared between mild and severe dengue patients. sVCAM-1 (**a**) were higher and sICAM-1 (**b**) were normal in dengue patients compared to healthy controls. Different trends of sVCAM-1 were observed between mild and severe patients

With regard to adhesion molecules, sVCAM-1 {*P* < 0.01 (Day 3–5), *P* < 0.01 (Day 6–7), *P* < 0.01 (Day 8–10), *P* = 0.036 (Day 14–17), Fig. 4a} were significantly higher and sICAM-1 (*P* > 0.05, Fig. 4b) were normal in dengue patients compared to healthy controls. Different trends of sVCAM-1 were observed between mild and severe patients as shown in Fig. 4a. Level of sVCAM-1 was displayed decreasing trend after day 6–7 in mild dengue patients while after day 8–10 in severe ones (Fig. 4a).

## Discussion

As we know, inflammation is an innate immune response to infection and supposed to be protective. However, nonresolving inflammation is a major trigger of disease and turns destructive [15]. Hence multiple mechanisms are evolved to resolve inflammation. Effector cells normally undergo apoptosis or differentiate to other phenotypes. Cells like macrophages could polarize from M1 towards M2 [16]. M1 macrophages play key roles in triggering inflammation via secreting pro-inflammatory factors, while M2 macrophages involve in immunosuppression and tissue repair by releasing series of anti-inflammatory mediators [17]. Discordance of those resolution mechanisms might result in excessive inflammation and detrimental outcomes. In this study, the expression profile of several important inflammation mediators, including TNF-α, IL-1α, IL-1, IFN-γ and IL-10, was investigated in dengue patients at different time points. Our results showed that there are significant differences between mild and severe patients. Those results provided solid evidence that inflammation played a pivotal role in determining the severity of dengue disease.

Once infected by DENV, the hosts immediately triggered an immune response to eliminate the virus. Similar to DENV, infections by other flaviviruses such as West Nile Virus (WNV) and Japanese encephalitis virus (JEV) also induce robust proinflammatory cytokine responses to cause immunopathology [18]. An appropriate inflammatory response is protective and essential for resistance to infection. Decreased or absent inflammatory response may lead to propagation of virus and progression of disease. However, excessive inflammatory response is believed to play a direct role in the pathogenesis of severe dengue disease [19, 20]. Studies have been reported that severe dengue patients display uncontrolled immune cell activation and increased inflammation mediators production. Elevated mediators change the activation status and function of the vascular endothelium and eventually lead to transient increase in vascular permeability [20, 21]. We also found high levels of inflammation mediators in severe patients in this study.

TNF-α, as a classic pro-inflammatory cytokine, stimulates the acute phase reaction. In this study elevated TNF-α was observed in all dengue patients but higher in severe cases. The important role of TNF-α in DENV infection has been reported in many previous studies [8, 22]. Although its inhibitory effect on DENV replication is benefit, TNF-α may also account for activation of vascular endothelial cell and increase vascular permeability. TNF-α has been associated with hemorrhagic manifestations of dengue [23]. Anti-TNF-α antibodies treatment of DENV-infected mouse has shown effects on preventing severe symptoms and reducing mortality [24]. However, inconsistency or no differences of TNF-α between different disease severity, even compared with healthy controls have also been reported in some studies [7]. This discrepancy could be due to TNF-α genetic polymorphisms or differences of study population. IL-1α, produced mainly by activated macrophages, is a member of IL-1 family like IL-1β. Increased IL-1β in severe patients has been reported when compared to mild cases [8]. However, Level of IL-1α in dengue patients has not been reported. In this study, increased IL-1α during the course of illness was observed. There were significant differences between mild and severe cases on day 8–10 of illness. IFN-γ has been reported to be associated with severity in many previous studies [25, 26]. IFN-γ displayed similar trend as IL-1α in this study. IL-10, as a central anti-inflammatory cytokine, suppresses the expression of pro-inflammatory cytokines, chemokines and adhesion molecules. Some study found that level of IL-10 was higher during febrile phase and correlate with the degree of plasma leakage [27, 28]. But other study reported that IL-10 peaked during defervescence as the body attempted to control the acute systemic inflammatory response [26]. In this study we observed that IL-10 peaked on day 6–7 in mild patients, while in severe cases on day 8–10 of illness. This discrepancy of IL-10 provided evidence of differences of inflammatory response between mild and severe patients. Chemokines also displayed differences between mild and severe dengue patients in this study. IL-8 is a chemokine produced mainly by macrophages. Similar to TNF-α, IL-8 is an innate immune mediator and mediates acute phase reaction. The effects on pathogenesis of severe dengue has been reported [26]. The trend of IL-8 in dengue patients was similar to TNF-α in this study. IP-10 is secreted by several cell types such as monocytes in response to IFN-γ and has important role in disease initiation and progression. Abnormal levels of IP-10 have been associated with inflammatory diseases [29]. In this study we observed significantly higher levels of IP-10 in dengue patients. The levels declined steadily in mild cases throughout the disease. However, the levels rose again in severe cases on day 8–10 of illness. This suggested that recurrent inflammatory response happened in severe cases. It can explain the trend of IL-10 at the same time point. Peaks of pro-inflammatory cytokines and peaks of anti-inflammatory cytokines coexist. MCP-1 is a potent monocytes chemotractant and has been reported that increased levels correlated with severe dengue symptoms [30]. The results in this study also proved that inflammation in severe dengue patients resolved differently from mild cases. However, lower levels of GRO-α and RANTES were observed in severe patients on day 6-7 than mild cases in this study. A recent study reported that RANTES levels were decreased in dengue patients and correlated strongly with platelets count and disease severity, similar to our results [9]. Lower levels of RANTES in dengue patients could be due to thrombocytopenia during defervescence particularly in severe cases. That was because GRO-α and RANTES were released by activated platelet [31]. Another study also proved that platelets from DENV infected patients secreted lower levels of RANTES *in vitro* than did platelets from healthy individuals [32]. The mechanism of decreased GRO-α may be the same as RANTES.

sVCAM-1 is released from the endothelial cell surface into the circulation upon endothelial activation and now proved to be a marker for endothelial injury under inflammatory processes [33]. Elevated levels of sVCAM-1 have been reported in dengue patients and associate with severity of disease [34]. In this study higher levels in severe dengue patients were observed than mild patients on day 8–10 of illness. Increased sVCAM-1 levels in all dengue patients indicated that abnormal function of vascular endothelial cells occurred after onset of illness as reported in another study. Evidence from serial ultrasound studies also indicated that plasma leakage actually started at initial stage of disease in all patients [35]. It is possible that most of patients experience some degree of plasma leakage and only a minority gets worse.

There is no specific antiviral treatment and vaccine currently available for DENV infection. Treatment is largely supportive to alleviate of symptoms and prevent shock by fluid resuscitation. Further investigations of the mechanisms of severe disease may provide insight into novel clinical management strategies. A number of studies have shown excessive inflammation mediators in severe dengue patients [36]. Inflammation mediators during DENV infection, like a double-edged sword, though anti-virus, may also account for increased vascular permeability that characterizes severe dengue [37, 38]. Defervescence, on around day 6–7 of dengue disease, is a watershed for disease progression that patients can either recover rapidly or progress to a severe life-threatening stage. As we observed in this study, most significant differences were noted on day 8–10 of illness between mild and severe dengue patients. That is, inflammation was very rapidly resolved in mild patients but not in severe cases. So suppression or pro-resolution of inflammation could be a potential therapeutic approach for treatment of severe dengue.

## Conclusion

Our results revealed the coexistence of excessive inflammatory response and slow resolution of inflammation in severe adult dengue patients. Thus, excessive inflammatory response or failed termination of inflammation usually contributes to the pathogenesis of severe dengue disease. Therefore, it is possible to introduce drugs with anti-inflammatory or pro-resolving effects to current therapeutic regimen, which might prevent severe disease development and decrease dengue mortality. Our study provides substantial evidence to support such hypothesis.

## Abbreviations

ALT, Alanine aminotransferase; AST, Aspartate aminotransferase; CK, Creatine kinase; DENV, Dengue Virus; GRO, Growth related oncogene; IFN-α, Interferon-α; IFN-γ, Interferon-γ; IL-10, Interleukin-10; IL-1RA, Interleuekin-1 receptor antagonists; IL-1α, Intereukin-1α; IL-6, Interleukin-6; IL-8, Interleukin-8; IP-10, Interferon-inducible protein-10; JEV, Japanese encephalitis virus; LDH, Lactate dehydrogenase; MCP-1, Monocyte chemotactic protein-1; MIF, Macrophage migration inhibitory factor; PLT, Platelet; RANTES, Regulated upon activation normal T-cell expressed and secreted; SD, Standard deviation; sICAM-1, soluble intercellular adhesion molecule-1; sVCAM-1, soluble vascular cell adhesion molecule-1; TNF-α, Tumor necrosis factor-α; WHO, World health organization; WNV, West nile virus
